# Reorganization and the A. M. A.

**Published:** 1904-12

**Authors:** 


### REORGANIZATION AND THE A. M. A.

THE attempt to bring the Medical Society of Virginia in line with the proposed reorganization or uniform plan of the A. M. A. was defeated at the annual meeting of the former body. The subject will be reopened at the meeting in April of the Medical Association of Georgia, and it is well for the members of the association to give the matter careful thought before committing themselves at that time irrevocably. The scheme briefly quoted may be stated in the words of the editor of the Virginia Medical Semi-Monthly, as follows:

"The plan ostensibly proposes primarily the organization of the doctors of each county into county medical societies. Delegates from these are to form the working part of each State society. The delegates from each State society—in the proportion of one delegate for every 500 members or a fraction thereof—form the House of Delegates of the American Medical Association. This House of Delegates of the National Association has supreme power to enact laws or to make rules for the government of the entire regular medical profession of the country. The idea is that in the organization of the county societies, if we take care of the pence the pounds will take care of themselves."

The pivotal idea in this plan is the construction of a system upon the unit represented by the county medical society. The whole plan hinges upon this. If there is no county medical society the physicians of that county can have no representation in the State or medical body, and no voice in matters that concern their welfare. Again, if there be a county organization, and any member of it has not paid his assessment of two dollars, the body to which he is allied loses its power of representation. Some of the counties of Georgia, according to Polk's directory, do not contain more than four or five regular physicians—not a very abundant material for organization purposes. If the county medical society is the supreme principle, is it not wise to consider the feasibility of constituting such bodies in our State before proceeding farther? No one disputes the need of organization of the medical profession. It is necessary for its welfare and protection, but no one desires that this organization shall smack of a trade union or lead to undemocratic centralization of power. These are some things that will have to be considered in making up one's mind as to the proper course to pursue when the question is presented for settlement at the next meeting of the association.

---

THE bulletin of Chicago's Health Department states: The dairy inspectors have inspected 659 dairies, 12,009 cows, representing the shipment of 2,486 eight-gallon cans of milk per day. Of these 659 dairies, 32 were in bad sanitary condition and 147 were feeding "wet malt." All of these were immediately notified that they could not ship milk to the city of Chicago, and the dealers to whom they shipped were also notified that they could not receive and sell this milk in the city. The milk from 11 other dairies was also excluded from the city because of bad sanitary conditions or diseased herds.

---

THE United States government is preparing to spend $600,000 in improving the reservation and park at Sulphur Springs, in the Chickasaw Nation, I. T., for the purpose of making a second Hot Springs similar to the famous Arkansas resort.

